# Immunotherapy for Glioblastoma: Current State, Challenges, and Future Perspectives

**DOI:** 10.3390/cancers12092334

**Published:** 2020-08-19

**Authors:** Minfeng Yang, In Young Oh, Arpan Mahanty, Wei-Lin Jin, Jung Sun Yoo

**Affiliations:** 1 Department of Health Technology and Informatics, The Hong Kong Polytechnic University, Kowloon, Hong Kong; minfeng.yang@connect.polyu.hk (M.Y.); i.y.oh@polyu.edu.hk (I.Y.O.); arpan.mahanty@connect.polyu.hk (A.M.); 2 Shanghai Engineering Center for Intelligent Diagnosis and Treatment Instrument, Department of Instrument Science and Engineering, School of Electronic, Information and Electrical Engineering, Shanghai Jiao Tong University, Shanghai 200240, China; 3 Key Laboratory for Thin Film and Microfabrication Technology of Ministry of Education, School of Electronic, Information and Electrical Engineering, Shanghai Jiao Tong University, Shanghai 200240, China

**Keywords:** glioblastoma, immune-checkpoint inhibitors, tumor microenvironment, tumor-associated macrophages and microglia, immune-related adverse events

## Abstract

Glioblastoma is the most lethal intracranial primary malignancy by no optimal treatment option. Cancer immunotherapy has achieved remarkable survival benefits against various advanced tumors, such as melanoma and non-small-cell lung cancer, thus triggering great interest as a new therapeutic strategy for glioblastoma. Moreover, the central nervous system has been rediscovered recently as a region for active immunosurveillance. There are vibrant investigations for successful glioblastoma immunotherapy despite the fact that initial clinical trial results are somewhat disappointing with unique challenges including T-cell dysfunction in the patients. This review will explore the potential of current immunotherapy modalities for glioblastoma treatment, especially focusing on major immune checkpoint inhibitors and the future strategies with novel targets and combo therapies. Immune-related adverse events and clinical challenges in glioblastoma immunotherapy are also summarized. Glioblastoma provides persistent difficulties for immunotherapy with a complex state of patients’ immune dysfunction and a variety of constraints in drug delivery to the central nervous system. However, rational design of combinational regimens and new focuses on myeloid cells and novel targets to circumvent current limitations hold promise to advent truly viable immunotherapy for glioblastoma.

## 1. Introduction

Glioblastoma (GBM) is the deadliest and most aggressive neuroepithelial cancer of the central nervous system (CNS) with an abysmal median survival of 14.6-month despite the multiple forms of intervention [1,2]. In the United States, the total annual incidence rate of glioma has been ~6 cases per 100,000 individuals, of which GBM accounts for about 50% of the cases, with a higher predominance in males [3]. Clinical studies have indicated that most of the GBM patients present an intact blood–brain barrier (BBB) for certain brain regions, capable of blocking the delivery of agents to cancer sites [4,5]. The BBB is considered to prevent diffusion of 98% of small-molecule and 100% of large-molecule agents into the brain from blood circulation [4,5]. Given the aggressive and heterogeneous nature of GBM and the blocking capability of BBB, a very limited number of medications for patients with GBM is available in clinics. In addition, due to the existence of other cellular and extracellular barriers, as well as the development of drug resistance over the treatment course, the efficacy of many current therapeutic approaches has been compromised. 

Currently available standards of care for GBM include maximal tumor resection followed by radiotherapy, chemotherapy, and corticosteroids, all of which have immune suppressive characteristics [6,7]. Unfortunately, complete surgical removal of the whole tumor is almost impossible due to their diffusive characteristics into normal brain tissue. Some reports indicated that ~65% of the post-surgery cases still showed residual tumor cells, which eventually contributed to a high relapse rate of GBM [8,9]. Therefore, GBM patients may undergo repeated surgical resection, radiotherapy, chemotherapy, or additional bevacizumab treatment. Eventually, most of the patients suffering from GBM will relapse despite an ample set of interventional approaches. According to the data from Surveillance and Epidemiology [10,11], the median overall survival (OS) of GBM patients was normally less than 2 years from the time of first progression or relapse. An international phase III randomized trial, conducted by the European Organization for Research and Treatment of Cancer/National Cancer Institute of Canada (EORTC/NCIC), has shown that the median OS of GBM patients who received radiotherapy and Temozolomide therapy remains poor (14.6 months) [1,2]. Moreover, Grossman and colleagues [7] found that the utilization of systemic chemotherapy and hyperfractionated radiation therapy with corticosteroids is likely to disable immune activity. Immune-suppressive characteristics, high toxicity, and lower OS of traditional care made a considerable number of GBM patients (~50%) not accept any second-line of anti-tumor treatment [10,11]. In addition, there is no evidence that traditional intervention can significantly impact the OS rate under a recurrence setting [12]. Accordingly, given the poor prognosis and limited therapy regimens for patients affected by GBM, there is an urgent need to develop novel therapeutic approaches. 

Cancer immunotherapy, particularly focused on immune-checkpoint inhibitors (ICIs), has achieved significant and promising clinical outcomes for a variety of cancer types, triggering tremendous interest as a new therapeutic strategy for GBM. Immunotherapeutic drugs do not kill tumors in a direct manner but, instead, they enhance the human immune system for more effective tumor death and long-lasting cancer remission with less adverse effects. There is a hope to see a similar effect by manipulating the GBM immune system with vibrant development of promising immunotherapies, particularly in light of the fact that the CNS does have active immune responses by recent findings. Herein, in this review, we highlight the diverse immunotherapeutic modalities, especially those major ICIs that have been applied to specifically target different molecules expressed on the surfaces of tumor or immune cells. In addition, we discuss some controversies and challenges related to the unique tumor microenvironment (TME), GBM biology, and immune-related adverse events. Compared with several other review papers, focus is given to new perspectives on nearly neglected myeloid cells and other emerged targets and combinational therapies to underpin and enhance existing cancer immunotherapies. Recent efforts for rational and sound design of clinical trials to increase the efficacy of anti-PD1 therapy were also included. Altogether, we aim to delineate a new blueprint for GBM immunotherapies by critically reviewing current state, addressing challenges, and providing novel perspectives for future direction. 

## 2. Immune Microenvironment of Glioblastoma

**Figure 1:**
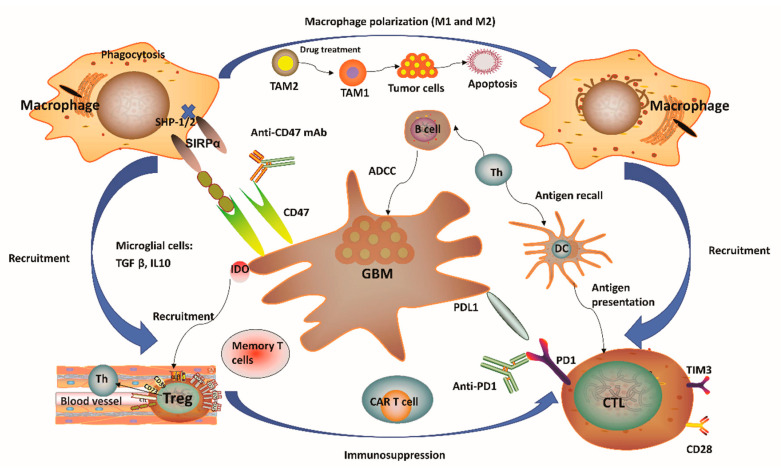
Immunity-related microenvironment of glioblastoma. (**1**) The immune microenvironment involving glioblastoma (GBM) is characterized by large amounts of CD8+ and CD4+ T cells, M1 and M2 polarized macrophages, microglia, and regulatory T (Treg) cells in addition to a limited number of natural killer (NK) cells. Tumor-associated macrophages and microglia (TAMs) have considerable plasticity toward anti-tumor M1 (inflammatory TAMs) and pro-tumor M2 (anti-inflammatory TAMs) phenotypes. Pharmacological strategies to re-educate tumorigenic M2 TAMs to tumoricidal M1 TAMs may help to relieve immune suppression in the tumor microenvironment (TME), as well as enhance the related anti-tumor activity. (**2**) GBM normally expressed high levels of immunosuppressive factors, such as programmed cell death 1 ligand 1 (PD-L1) and indolamine 2,3-dioxygenase (IDO), while limiting the presentation of antigens by decreasing major histocompatibility complex (MHC) presentation. The application of IDO inhibitors has effects on Treg cell accumulation. (**3**) CD47 is highly expressed in various types of tumors. Signal regulatory protein α (SIRPα) is an inhibitory receptor expressed on macrophages and other myeloid immune cells. Upon CD47 binding to SIRPα, src homology 2 domain-containing protein tyrosine phosphatase 1 (SHP-1) and SHP-2 phosphatases are activated to further abrogate phagocytosis via downstream mediators. Disruption of the CD47/SIRPα axis using anti-CD47 antibody (CD47 Ab) can interrupt the inhibitory signaling mediated by SIRPα, thereby promoting phagocytosis of tumor cells. (**4**) T-cell immunoglobulin and mucin domain-containing protein-3 (TIM3) is a strong negative regulator of lymphocyte function and survival, acting as a marker of CD4+ and CD8+ T-cell exhaustion similarly to programmed cell death 1 (PD-1). It has been verified that the co-expression of PD-1 and TIM3 in lymphocytes is positively correlated with the tumor grade, but it is negatively correlated with progression-free survival (PFS) in different types of tumors including GBM. (**5**) In the context of microglial cells, these often secrete transforming growth factor β (TGFβ) and/or interleukin 10 (IL-10) to decrease the amount of myeloid and/or lymphoid immune cells, resulting in a systemic immunosuppression and immune evasion of GBM cells. Th, helper T cell; ADCC, antibody-dependent-cell-mediated cytotoxicity; Treg, regulatory T cell; CTL, cytotoxic T lymphocyte; CAR T, chimeric antigen receptor T cell; DC, dendritic cell.

It has been regarded that the CNS lacks dedicated lymphatic channels for a long time. The CNS was considered as an immune privileged system, devoid of any immune cells. This overstated historical notion was mainly based on the experimental data reported by Peter Medawar, where foreign grafts transplanted into the brains of rodents did not induce any immune response and the same foreign grafts transplanted into other tissues or organs were rejected [13,14,15,16]. However, this perception has been challenged recently since several studies showed vigorous immunosurveillance and meaningful immune response in the CNS [17,18]. For example, the discovery of a novel route of lymphatic-based channels, reported by Louveau and colleagues [17] in 2015 and the findings of robust immune responses in multiple inflammatory conditions [18] have both demonstrated the CNS as a region for active immunosurveillance. Such findings prompted an increase in studies for the feasibility of cancer immunotherapy towards brain tumors. Although immunotherapy holds great potential for treatment of malignant GBM, unique GBM-associated immune suppression and immune escape still provide challenges to generate efficient anti-tumor responses [16,19]. GBM can form a highly immunosuppressive milieu, mediated by distinct immune or tumor cells (Figure 1). Tumor cells normally express plenty of immunosuppressive factors, such as programmed cell death 1 ligand 1 (PD-L1) and indolamine 2,3-dioxygenase (IDO), while reducing antigen presentation by diminishing major histocompatibility complex (MHC) expression [16]. Notably, gliomas produce IDO, whose function relates to the recruitment of regulatory T (Treg) cells and the inhibition of effector T cells through tryptophan depletion [16,20]. In the context of microglial cells, these often secrete transforming growth factor β (TGFβ) and/or interleukin 10 (IL-10) to decrease the amount of myeloid and lymphoid immune cells to boost systemic immunosuppression [16,21,22] (Figure 1). The lymphoid compartment also mediates immunosuppressive effects with Treg cells through upregulation of different soluble factors and some immune-checkpoint molecules [16]. These immunosuppressive factors may ultimately block T-cell proliferation and activation. One unique factor of GBM is its relatively low tumor mutational burden (TMB) which reduces the responding T cell clones resulting in poor adaptive immunity [23,24,25]. High TMB often suggested as a reliable biomarker for ICIs [23,24,25]. Other variables, including chemotherapy, corticosteroids, and patient age-related factors may also lead to immunosuppression in GBM patients [6,7,22]. Overall, GBM is considered as a highly immunosuppressive CNS-related tumor.

One distinctive aspect of the brain’s microenvironment is related to the bulk of myeloid cell population, which is capable of manipulating the immune microenvironment and GBM progression by producing immunosuppressive and anti-inflammatory cytokines and growth factors, as well as promoting T-cell apoptosis, thus suggesting a new strategy for immunotherapy [26,27]. A considerable population of brain myeloid cells are microglia, which are equivalent to macrophages from other tissues [28]. In the absence of any inflammatory stimulation, the microglia normally arise from the yolk sac and are maintained by continuous replication during our whole life [29]. Upon pro-inflammatory stimulation in the GBM tissue, microglial cells may undergo significant phenotypic changes, while extensive additional macrophages can also be recruited from peripheral monocytes into the tumor site [30,31,32]. Notably, the GBM microenvironment has a surprisingly high composition of tumor-associated macrophages and microglia (TAMs), ranging between 30 and 50% [33] of tumor mass. Such a percentage of TAMs is much higher than the ones observed in other major malignancies such as melanoma [34]. One notable feature of TAMs is that they have considerable plasticity toward anti-tumor M1 (inflammatory TAMs) and pro-tumor M2 (anti-inflammatory TAMs) phenotypes (Figure 1). Redirecting TAMs from immunoinhibitory M2 to immunostimulatory M1 phenotype is a promising approach to elicit an immune response and to inhibit GBM progression since this can reduce immunosuppressive restrains and thus boost immunity driven by cytotoxic T lymphocytes (CTLs) [27,35]. More recently, research evidence has indicated that pharmacological inhibition provided by certain soluble factors, such as colony-stimulating factor-1 receptor, can dramatically decrease M2 polarization and significantly improve OS [36]. Moreover, several reports have confirmed a strong association between the survival of high-grade glioma patients and M1 or M2 polarization. For instance, M1 polarization has been positively correlated with improved patient survival [37]. In contrast, M2 polarization (assessed by F11R marker) has been negatively correlated with patient survival [38]. Therefore, strategies to target TAMs have emerged as alternate routes for GBM therapy [39]. In this sense, a number of studies have pursued ways to (i) inhibit monocyte recruitment into the CNS, (ii) deplete M2 TAMs, and (iii) reprogram tumorigenic M2 to M1 phenotype [40]. One recent report has also demonstrated TAM-mediated resistance of programmed cell death 1 (PD-1) immunotherapy, thus providing a strong rationale towards TAM targeting as a reliable approach to enhance PD-1-inhibitor treatment response [41]. Of note, TAM-targeted immunotherapy has received particular attention in recent years although investigations related to this promising therapeutic area are still in progress.

## 3. Overview of Current Immunotherapy Modalities for Glioblastoma

**Figure 2:**
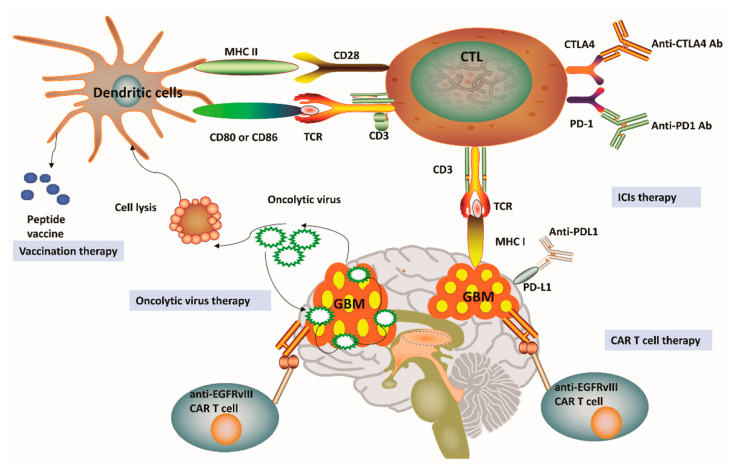
Current immunotherapy strategies for glioblastoma. (**1**) Vaccines for glioblastoma (GBM) treatment have been relied on dendritic cell (DC)-mediated presentation of GBM-related antigens and peptides for T-cell activation in the adaptive immune system. (**2**) The immunosuppression status of cytotoxic T lymphocytes (CTLs) can be relieved by the application of immune-checkpoint inhibitors (ICIs), including anti-programmed cell death protein 1 (PD-1), anti-cytotoxic T lymphocyte protein 4 (CTLA-4) and anti-programmed cell death 1 ligand 1 (PD-L1). (**3**) Genetically engineered chimeric antigen receptor (CAR) T cells can generate artificial T-cell receptors with high affinity to cancer-specific antigens. (**4**) Genetic engineering has also been applied in oncolytic viral treatment to medicate cancer cell lysis and promote tumor necrosis. MHC, Major histocompatibility complex; TCR, T-cell receptor; EGFRvIII, Epidermal growth factor receptor variant III.

As a paradigm shift in cancer treatment, immunotherapy has recently gained enormous attention and also achieved a rapid expansion in the context of GBM. Immunotherapy approaches for GBM have been focused on ICIs, oncolytic viruses, chimeric T-cell receptors, and dendritic cell (DC) vaccines [16,21]. Figure 2 outlines four distinct immunotherapy modalities available for GBM. We can notice that a successful vaccine for GBM treatment depends on DC-mediated presentation of GBM-related antigens as well as peptides for T-cell activation in the adaptive immune system. Among the pathways involved in these processes, one is related to the combination of T-cell receptors and MHC, while another pathway involves the interaction between CD80/CD86 and CD28. Cytotoxic T lymphocytes (CTLs) can be subsequently activated to kill GBM cells having specific antigens for MHC I presentation [16,42]. In general, tumor cells avoid this disruption by upregulating PD-L1, which binds to its complementary receptor, PD-1 along the T-cell surface to further inhibit the activation of CTLs [42]. We can utilize different approaches of immune-checkpoint blockage to effectively prevent the interaction between PD-L1 and PD-1 in GBM. However, a phase III trial result to compare therapeutic efficacy of nivolumab and bevacizumab in recurrent GBM was disappointing with no improvement in OS (Clinical trial identifier: NCT02017717, Table 1). Cytotoxic T lymphocyte protein 4 (CTLA-4) is another important immune regulatory molecule that binds to CD80 or CD86 and inhibits their combination with CD28 to prevent T-cell activation [43]. Epidermal growth factor receptor variant III (EGFRvIII), IL-13 receptor subunit-α2 (IL-13R α2), and human epidemic growth factor receptor 2 (HER2) are expressed on the surface of GBM cells and may also be targeted by a genetically modified chimeric antigen receptor (CAR) T cell to promote GBM cell death [16,21,42]. Given the promising role of cancer immunotherapies towards GBM pathophysiology, a substantial number of clinical trials have been performed or planned to explore the potential roles and efficacy of targeting these three antigens [16,21,42] (Table 1). These clinical trials have demonstrated feasibility, safety, and efficacy of CAR T cell therapy for GBM. For example, treatment constituted of virus-specific T cells (VSTs) expressing HER2-specific CAR (HER2-CAR VST)in progressive GBM patients resulted in an median OS of up to 11.1 months from the first T cell infusion and 24.5 months from the first diagnosis (Clinical trial identifier: NCT01109095, Table 1).Interestingly, genetic engineering has also been applied in oncolytic viral treatment to produce viruses that may infect tumor cells, trigger tumor cell lysis, and hijack tumor cell replication, which ultimately leads to tumor cell death [16,21] (Table 1). This particular treatment has enabled the breakage of shackles from many tumors and also triggered a higher immune backlash, thus shifting GBM from cold to hot tumor types [16,21]. Promisingly, data from phase II trial have verified the high clinical response in GBM patients after intratumoral inoculation of Polio/Rhinovirus Recombinant (PVSRIPO), with an increase in OS up to 12.5 months from the time of inoculation and higher survival rate at 24 and 36 months over historical controls (Clinical trial identifier: NCT01491893, Table 1). These results show that oncolytic-based therapy has a high potential to improve OS and quality of life for patients affected by GBM [42]. Although oncolytic-based therapies may provide significant immunostimulatory effects, including the depletion of regulatory T cells, the induction of immunogenic cell death, and abscopal effects, these therapeutic approaches still carry some intrinsic limitations. For instance, pro-inflammatory responses caused by oncolytic viruses may potentially limit the application of oncolytic viruses as a single-modality immunotherapy [42]. Besides, CAR T-cell treatment for GBM relies on the identification of stably expressed and sufficient tumor-related antigens, which might eventually limit the clinical application of this therapy [21,42]. Considering the highly heterogeneous characteristics of GBM, one could postulate that targeting one antigen in GBM might not be sufficient to eradicate all the GBM cells. Overall, these obstacles have promoted the development of alternate immunotherapy modalities, which may better recapitulate tumor immunology with improved accuracy.

## 4. Immune-Checkpoint Inhibitors

During recent decades, ICIs have been emerged as a promising alternative for cancer treatment. Nowadays, the success of immunotherapy against various types of cancers based on the stimulation of the host immune system to kill “self” tumors has spurred the identification of novel immunotherapeutic targets. So far, most of the treatments have focused on stimulating the adaptive immune system to kill tumor cells, including approaches targeting CTLA4, PD-1 or PD-L1 [43,67]. These therapeutic agents have shown great benefits for patients with advanced cancers [68]. As a result, ICIs such as Ipilimumab, Nivolumab, and pembrolizumab were approved since the 2010s by the Food and Drug Administration (FDA) for the treatment of malignant cancers, such as non-small-cell lung carcinoma (NSCLC), melanoma, lymphoma, renal, liver, bladder, and head and neck cancers [68,69]. Those agents facilitate an effective antineoplastic immune response by suppressing co-inhibitory receptors and pathways that are activated by tumors to suppress T-cells’ response against tumour cells. Of particular relevance is the verification of ICIs that can triger a durable and deep remission. Despite the concern of therapeutic-related toxicity, the use of ICIs can be taken into consideration because, in most cases, these side effects are still manageable. According to the expansion of immune-checkpoint blockade as a therapeutic strategy, synergistic and antagonistic interactions between distinct ICIs have been largely increased. Some issues remain to be elucidated, including the proper utilization and/or combination of different ICIs in GBM as well as the outcome of their applications towards positive or negative interactions.

### 4.1. Cytotoxic T Lymphocyte Protein 4 (CTLA-4)

CTLA-4 (CD152) is the first immune-checkpoint molecule identified as a main effector capable of impeding immune response and, therefore, targeted for therapeutic purposes. The presence of CTLA-4 in the T-cell surface is normally combined with the ligands CD80 or CD86, which are expressed by antigen-presenting cells (APC) to inhibit co-stimulators in T cell-related pathways [69]. In 2011, the CTLA-4 targeted humanized antibodies, Ipilimumab and Tremeumumab (for melanoma and mesothelioma treatment, respectively) were approved by the FDA and European Medicines Agency (EMA) [69]. In GBM patients, the expression of CTLA-4 in CD4+ or CD8+ cells is correlated with a poor OS of patients affected by GBM [70].

### 4.2. Programmed Cell Death 1 (PD-1) and Programmed Cell Death 1 Ligand 1 (PD-L1)

PD-1 is expressed on the surface of T cells. Interestingly, both PD-1 and its ligand (PD-L1) are aberrantly expressed on the surface of cancer cells and APCs with tumor progression. These two molecules have been considered the most notable immune-checkpoint targets identified so far (Figure 2). The combination of PD-1 and PD-L1 is capable of inhibiting early CTL activation, abolishing their cytotoxic activity toward cancer cells as well as reducing the production of inflammatory cytokines [71,72]. Some studies have shown the high expression of PD-L1 on GBM cell surface acts as a predictive factor correlated with a poor prognosis in patients [73,74]. However, as determined in some failed trials of checkmate-143, only 27% of patients presented PD-L1 expression level >10%, while 32% of GBM cases expressed PD-L1 in <1% of cancer cells [75]. Another essential predictor is TMB, which augments the amount of neoantigens and triggers a robust antitumor response. A high TMB in one GBM patient with mutations in the gene coding DNA polymerase epsilon (POLE) led to a systemic response to pembrolizumab (PD1 inhibitor), thus supporting this hypothesis [76]. Nevertheless, GBM normally has a low mutation rate and limited infiltration of T cells, which likely diminishes the efficiency of ICIs [23].

Monoclonal antibodies (mAbs) against PD-1 (Nivolumab, Pembrolizumab, and Cemiplimab) and PD-L1 (Atezolizumab, Durvalumab, and Avelumab) were approved by FDA in 2014, 2016 and 2017, respectively [70,76]. By overcoming PD-1-mediated inhibition of antitumor immune response, the therapeutic use of these antibodies has been extended to 17 distinct types of advanced and unresectable tumors [70,76]. The efficiency of PD-1/PD-L1 on T-cell priming, effector function, and immune exhaustion has provoked intense investigation in the field of GBM therapeutics. However, one retrospective study indicated that a single PD-1 blocking agent with better tolerance (i.e., Pembrolizumab) was unable to improve the OS of patients who suffered from recurrent high-grade glioblastoma (HCG), which represented an OS of 4 months [47] (Table 1). The reason for the failure of single ICI treatment seems to be complex, but it appears to be highly associated with the insufficient access of ICIs to GBM through the BBB. Although combinational therapy regimens are more likely to offer advantages over single ICIs in terms of the OS of GBM, the careful design of clinical trials is critical, given that different combinational therapies or experimental setups may influence the therapeutic response in GBM patients. For example, recruited participants in some clinical trials have received high concentrations of corticosteroids, which dramatically interrupt the therapeutic efficiency of ICIs and, consequently, lead to a poor therapeutic outcome.

### 4.3. CD47: A Newly Emerged Immune-Checkpoint Inhibitor for Glioblastoma

CD47, a member of the immunoglobulin superfamily, is known to play an antiphagocytic role in the TME and to contribute with tumor recurrence. There is increasing evidence demonstrating that the binding of CD47 to signal regulatory protein α (SIRPα) triggers a signaling cascade that restrains macrophage activation (Figure 1) [77]. Interferencing CD47/SIRPα axis using anti-CD47 antibodies has been effective in inhibiting the growth of certain solid tumors including melanoma, lung cancer and leiomyosarcoma (Figure 1) [78,79,80,81]. Based on the benefits achieved for the treatment of other malignancies, CD47-targeting antibody and human (or murine) SIRPα-Fc have been used to access the anti-tumor effect of GBM by blocking CD47-SIRPα axis in vitro and in vivo. Notably, more recent efforts have sought to block CD47/SIRPα axis by using two types of anti-CD47 antibodies (Hu5F9-G4 and MIAP301) [77,78,79,80,81,82,83,84]. These antibodies were effective in the treatment of human patient-derived primary xenograft models with malignant brain tumors by promoting pro-inflammatory environment through innate immune surveillance [77]. Interestingly, an increasing amount of evidence has recently indicated that underappreciated adaptive immune responses to anti-CD47 therapy may reflect into a prolonged OS in addition to innate macrophage responses in the TME [82]. In view of the dynamic characteristics of macrophages, the anti-CD47 therapeutic regimen may trigger the polarization of immunoinhibitory phenotype M2 macrophage towards an immunostimulatory M1 phenotype in GBM. Therefore, CD47 blockage represents a promising avenue to create pro-inflammation TME that could augment anti-tumor response by enhancing M1 macrophage response [83] which may be particularly effective for GBM treatment considering the surprisingly high composition of TAMs, 30–50% of tumor mass.

Some preclinical studies have supported the utilization and suitability of blocking CD47/SIRPα for GBM treatment. However, the efficacy of single anti-CD47 therapy is limited and it can only eradicate tumors partially [84]. The mechanism underlying these observations may involve the ubiquitous expression of CD47 throughout the human body, where a huge ‘antigen sink’ might be needed to lump the loading dose and high frequency of agent administration to enable the therapeutic blockage of CD47. There are substantial ongoing investigations to improve the treatment efficiency of CD47. For instance, one recent study showed that the anti-tumor efficiency of CD47/SIRPα blockades can be reinforced upon combination with CTLA-4, PD-L1 or other ICI blockers [82]. To verify this hypothesis, Sockolosky J.T. et al. has designed a nanobody, which is able to antagonize the murine CD47 [82]. In this study, the agent itself did not show significant anti-tumor effect but, when combined with a tumor-opsonizing antibody or anti-PD-L1, it was able to produce a systematic anti-tumor response [82].

### 4.4. T-Cell Immunoglobulin and Mucin Domain-Containing Protein-3 (TIM3) and Idolamine 2, 3-Dioxygenase (IDO)

**Figure 3:**
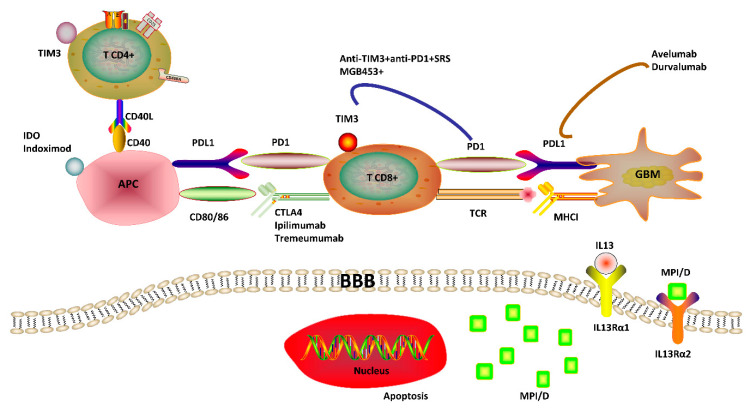
Immunotherapy with Controlled Nano-drug Delivery System for Glioblastoma. (**1**) Doxorubicin (DOX)-loaded mesoporous silica nanoparticle (MSN) coated with IL13Rα2-targeted peptide (IP) using polyethylene glycol (PEG) (MPI/D) is a promising vehicle for the targeted delivery of DOX to glioblastoma (GBM) in vitro and in vivo. (**2**) Interleukin-13 receptor subunit alpha-2 (IL13Rα2) has a function of restraining the Janus kinase (JAK)-signal transducer and activator of transcription (STAT) pathway activation by inhibiting IL13-targeted IL13Rα1, thus reducing the expression of tumor protein p63 (p63) and STAT6, which was already proven to be a hindrance of tumor formation. TIM3, T-cell immunoglobulin and mucin domain-containing protein-3; IDO, Idolamine 2, 3-dioxygenase; APC, Antigen-presenting cell; PD1, Programmed cell death 1; PD-L1, Programmed cell death 1 ligand 1; CTLA-4, Cytotoxic T lymphocyte protein 4; MHC I, Major histocompatibility complex I.

TIM3 is an immune regulatory molecule, expressed by CD4+ and CD8+ T cells, that participates in immune suppression and promotes tumor escape through exhaustion of T lymphocytes [85]. In GBM patients, TIM3 overexpression is correlated with higher level of malignancy (higher tumor grade, lower Kamofsky). Thus, TIM3 has been recognized as a strong negative prognosis indicator of GBM (Figure 3) [85,86,87]. 

IDO is not a standard immune-checkpoint molecule with lack of receptor capacity. However, this enzyme also functions as a suppressive molecule on CTL activation and natural killer (NK) cell function [88,89,90]. Similar to TIM3, IDO overexpression is related to poor outcome in GBM patients (Figure 3) [90]. Targeting IDO with Epacadostat or Indoximod has been a successful experimental strategy using in vivo models [88,89,90]. Following the benefits reported in preclinical models, several clinical studies are currently underway with Indoximod in combination with radiotherapy and chemotherapy and/or PD-1 inhibitor (Clinical trial identifier: NCT04047706, NCT02502708, Table 1).

## 5. Novel and Combinatorial Therapies: Preclinical Findings

As the immune-checkpoint blockade strategy becomes more widely available, both synergistic and antagonistic interactions between current standard GBM therapies and ICIs have become a focus of active investigation for better treatment outcome. However, a number of questions remains to be answered, including how to combine different ICIs with conventional therapies (radiotherapy, chemotherapy, and Bevacizumab) and whether the application of combinatorial agents involves positive or negative interactions. In fact, combinatorial GBM treatments will require more careful design for some variables such as mode of delivery, timing, and potential concern for increased toxicity. Moreover, given the expanding number of immunological targets involved in GBM, as well as the extensive list of immunotherapeutic agents under development, the number of available therapeutic combinations is prohibitively large for random testing. Therefore, the rational design of combinational regimens is essential to establish optimal therapeutic strategies. In addition to combinatorial therapy approaches, the focus has been also given to the development of novel therapies such as nanomaterial-based therapy, myeloid cells based strategy, new molecular targeting, and modification of treatment regimens to overcome paramount challenges of GBM treatment with immunotherapy.

### 5.1. Dual Treatment of PD-1 and TIM3 Blockades with Stereotactic Radiosurgery (SRS)

TIM3 expression in T lymphocytes has been suspected as mechanism of adaptive resistance to anti-PD-1 therapy [91]. In the murine glioma model studies, the upregulation of TIM3 in PD-1 antibody bound T cells was reported after failure of PD-1 blockade treatment [91] and the increase in exhausted PD-1 + TIM3 + T cells was observed in a time-dependent manner with tumor progression [92]. Having determined the co-expression of TIM3 and PD-1 in the T cells, there have been vibrant preclinical investigations on anti-tumor effects of dual PD-1/TIM3 blockade treatment for improved survival [91,92,93]. Following promising preclinical results, a clinical trial to evaluate the combinational use of stereotactic radiosurgery (SRS) with MBG453 (anti-TIM3 antibody) and Spartalizumab (anti-PD-1 antibody) is currently underway (Clinical trial identifier: NCT03961971, Table 1, Figure 3). The rationale of this combination strategy is based on the initial application of local radiotherapy to drive the release of tumor antigens, followed by immunotherapy, to ultimately promote an anti-tumor immune response. SRS has been proven to synergize anti-PD-1 therapy in orthotopic mouse GBM models, by leading to an increase in the amount of CD8+ T cells expressing interferon gamma (IFNγ) as well as a decrease of tumor-infiltrating T reg cells, when compared with a single treatment with SRS or anti-PD-1 therapy [92]. A persistent OS benefit was reported in mice with combinational treatment, and evidence of immune memory was observed by a lack of cancer cell engraftment upon the re-challenge of mice previously treated with anti-PD-1 and SRS [92]. This paradigm has been mainly predicated on the combination of immunotherapy with stereotactic radiosurgery (NCT04225039) [94] as well as laser ablation (NCT02311582) [95], and oncolytic viral treatments (NCT02798406) [53].

### 5.2. Immunotherapy with Controlled Nano-Drug Delivery System

One major obstacle in GBM therapeutics is that most of the affected patients still present brain regions with an intact BBB, thus restricting drug delivery and greatly compromising the immunological targeting [96,97,98]. One viable route to address this issue is to use local chemotherapy together which harbors synergistic activity with immunotherapy. However, some evidences have indicated that direct and local intracranial delivery could induce several side effects such as infection, edema, and backflow along the catheter. Notably, there has been intense interest in exploiting nanotechnologies that could be applicable for GBM treatment to overcome the constraints of BBB [99,100,101]. Nanotherapeutics combining nanomaterials and GBM targeting molecules not only improve therapeutic efficacy but also circumvent limitations of conventional chemotherapies such as limited permeability, selectivity, and retention [101]. Recently, one research has shown that a mesoporous silica nanoparticle (MSN)-based vehicle coated with interleukin-13 receptor subunit alpha-2 (IL13Rα2)-targeted peptide (IP) using polyethylene glycol (PEG) (namely MSN-PEG-IP, MPI) could be utilized as an effective drug delivery system for GBM therapy (Figure 3). In this work, doxorubicin (DOX)-loaded MPI (MPI/D) has successfully transmitted DOX to GBM cells in vitro and in vivo without affecting normal brain tissues and significantly improve the OS for GBM models [101]. Such significant enhancement on the cellular uptake of DOX in glioma may serve as a potential GBM-targeted drug delivery system [101]. Moreover, another promising finding in this research is that MPI not only delivers DOX to GBM in a targeted manner but also occupies IL13Rα2 and then promotes the binding of IL-13 to IL13Rα1, thus activating the Janus kinase (JAK)-signal transducer and activator of transcription (STAT) pathway to trigger an anti-tumor effect [101] (Figure 3).

### 5.3. A New Perspective on PD-1 Targeted Immune-Checkpoint Inhibitors for Myeloid Cells 

Among the array of cancer-related immunotherapies currently available, the immune-checkpoint molecule PD-1 has revolutionized the care of patients with multiple advanced cancers. The PD-1 mAb is believed to interrupt the engagement of PD-1 with its inhibitory ligands, spurring CTL-mediated cancer elimination [71]. It is worth noting that previous work has mainly focused on the anti-tumor role of PD-1 mAb in T cells, but the role of PD-1 mAb in myeloid cells remains unclear. Recently, Strauss and colleagues conducted an exploratory study verifying that the expression of PD-1 in myeloid cells can restrain host immunity against cancers and, therefore, it could be also a good target of anti-PD-1 ICIs [102]. Moreover, researchers generated new mouse models (PD-1f/fLysMcre mice and PD-1f/fCD4cre mice) upon the ablation of PD-1 in myeloid or T cells by conditionally knocking out the respective Pdcd1 alleles. Surprisingly, the inhibition of tumor growth in PD-1f/fLysMcre mice with PD-1 deleted myeloid cells was similar to the one in the mice with complete PD-1 deletion (Pdcd1−/−). Other results have also demonstrated that the anti-tumor efficiency of myeloid-specific deletion of PD-1 (PD-1f/fLysMcre mice) was much higher than the one of T-cell-specific PD-1 ablation (PD-1f/fCD4cre mice) [102]. Myeloid-specific PD-1 ablation induced altered signaling and metabolic reprogramming with enhanced differentiation of effector myeloid cells and emergency myelopoiesis driven synthesis of cholesterol, which is important for the differentiation of inflammatory macrophages and DCs. As a result, the number of immunosuppressive myeloid-derived suppressor cells (MDSCs) was decreased and the amount of differentiated and inflammatory effector myeloid cells was increased. Moreover, the ablation of PD-1 in myeloid cells triggers the increase of effector memory T cells, which have a distinctive function assisting anti-tumor activities. Together, these findings shed new light on the key mechanism of antitumor effect by PD-1 blockade mediated via myeloid cells.

### 5.4. CD73 Targeting Approach: An Efficient Route to Improve Outcome of Glioblastoma Treatment

Recently, many studies have indicated that the response to ICIs is mostly cancer type dependent. Although the underlying mechanism for those disparities has not been fully understood, current studies have indicated that different immune infiltrates including tumor-infiltrating lymphocytes (TILs) may be correlated with different clinical outcome of specific tumors. More recently, Goswami and colleagues performed comprehensive mass cytometry and single-cell RNA sequencing in patients with NSCLC, prostate cancer, renal cell carcinoma, colorectal cancer, and GBM. The results show that CD73hi macrophages were overexpressed in GBM, which has multiple drug resistance to ICIs. Moreover, MARCO (macrophage receptor with collagenase structure), TGFβ, and multiple SIGLEC (sialic acid-binding immunoglobulin-type lectins) genes are highly expressed on CD73hi cells [103]. According to TCGA (The Cancer Genome Atlas) data, CD73hi gene features are correlated with lower OS rates of GBM patients. Moreover, analysis of GBM samples from patients treated with anti-PD-1 antibodies indicates that this therapy does not cause any significant TME alterations. Such patients still have a high population of CD73hi cells capable of inhibiting T-cell infiltration that lead to poor clinical response to ICIs. Upon knocking out Nt5e (which encodes CD73) in a GBM mouse model, the efficiency of a combinational treatment with anti-CTLA4 and anti–PD-1 was elevated. In particular, the improved efficacy was correlated with obvious enhancement in macrophage polarization toward M1 population and infiltration of T cells in *Nt5e* knockout mice. Taken together, a variety of evidence has indicated that therapies involving anti-CD73 may function as an efficient approach to improve OS in patients affected by GBM [103].

### 5.5. Neoadjuvant PD-1 Blockade Treatment to Improve Immunotherapy Efficacy

Despite numerous preclinical successes of anti-PD1 therapy, a recent clinical trial of PD-1 blockade in recurrent GBM has indicated that only 8% patients showed clinically meaningful response [104]. To improve such disappointing therapeutic efficacy, very recent ongoing clinical studies modified treatment regimens by utilizing neoadjuvant checkpoint blockade treatment in addition to standard adjuvant therapy against GBM [105]. Neoadjuvant immunotherapy has been applied in other types of cancers such as lung cancer [106] to boost systemic immunity against tumor antigens, wiping out micro-metastatic cancer deposits that might be the source of postsurgical relapse. In regard to adjuvant treatment that may directly eliminate micro-metastatic tumors upon surgical resection, the application of neoadjuvant PD-1 blockade while the primary tumor is in place may leverage a higher level of endogenous tumor antigen release in the primary tumor to further promote T-cell priming. In particular, immunostimulatory therapy following surgery resection might be more beneficial to reduce residual disease burden and thus improve the likelihood of clinical benefits [106]. To address whether neoadjuvant PD-1 blocking could dramatically change the functional immune landscape and then improve OS in recurrent GBM patients, the Ivy Foundation Early Phase Clinical Trials Consortium has recently conducted a multi-center, open-labeled pilot randomized clinical trial to assess immune response and OS following neoadjuvant as well as adjuvant treatment with pembrolizumab [105]. Respective results have indicated that neoadjuvant PD-1 blocking may significantly down-regulate the expression of genes related to cell cycle but, at the same time, up-regulate the expression of T cell- and interferon-γ-related genes, which are rarely observed in patients who received adjuvant treatment alone. Moreover, decreased PD-1 expression on T cells, reduced monocytic population, the focal induction of PD-1 in TME and strengthened clonal expansion of T cells are frequently observed in neoadjuvant setting when compared with the adjuvant group. Most importantly, treatment with neoadjuvant Pembrolizumab has led to a statistically significant improvement in OS (13.7 months) and PFS (3.3 months), in comparison to the lower OS (7.5 months) and PFS (2.4 months) that was achieved in the adjuvant cohort [105]. Another single-arm phase II clinical trial (Clinical trial identifier: NCT02550249, Table 1) with neoadjuvant nivolumab was conducted [107]. A total of 30 patients involved in this clinical trial received pre- and post-Nivolumab during GBM progression or until reaching intolerable toxicity. As expected, augmented chemokine expression, enhanced T-cell receptor clonal diversity as well as increased immune cell infiltration were observed in a cohort of GBM patients who received the neoadjuvant Nivolumab, thus promoting a local immunomodulatory therapeutic effect [107]. In particular, even though disease relapse was inevitable and no apparent clinical benefits were observed following salvage surgery resection, 2 of 3 GBM patients who received Nivolumab before and after surgery remained disease-free for further 33 and 38 months. These two investigations indicate that neoadjuvant anti-PD-1 therapy may trigger an enhanced local and systemic immune response and significantly improve the median OS and PFS when compared with adjuvant therapy alone, thus acting as a more efficient approach for GBM therapy [107]. 

## 6. Clinical Management of Immune-Related Adverse Events Induced by Immune-Checkpoint Inhibitors

Despite the clinical benefits acquired by ICI-based treatments, early clinical trials have suggested that ICIs may lead to immune-related adverse events (irAEs) [108]. In fact, ICIs are not perfectly targeted to cancer-specific T cells only, which means that the ability of ICIs to elicit anti-tumor response may be accompanied with activation of non-specific immune reactions against antigens expressed by normal tissues [109]. It has been reported that irAEs may occur in a variety of organs after ICI treatment, frequently leading to skin conditions such as pruritus, mucositis, and maculopapular rash. Surprisingly, up to 15% of patients receiving anti-PD-1 or PD-L1 can ultimately develop immune-related rashes [110]. A recent case study has shown that the combinational therapy of betamethasone and oral prednisone can significantly relieve the cutaneous toxicity with maculopapular rash, thus allowing the ICI treatment to resume subsequently after symptom relief (Table 2) [111,112]. Likewise, gastrointestinal discomfort (in the form of immune-mediated colitis and diarrhea) has been commonly observed, with up to 40% of patients treated with Ipilimumab experiencing this adverse effect [110]. Less common irAEs include pneumonia, endocrine diseases, and hepatotoxicity. Regarding pneumonia, patients receiving Durvalumab are more likely to be develop irAEs, whereas these symptoms can be mostly managed by corticosteroids according to the toxicity level of ICI-related pneumonitis (Table 2) [110]. In a limited number of cases, blood abnormalities, uveitis, cardiovascular toxicity, neurotoxicity, ocular manifestations, and renal toxicity have also been reported (Table 2). In general, the incidence of irAE due to CTLA-4 blocking is much higher than that of PD-1 or PD-L1 immune-checkpoint inhibition (Table 2) [111]. Moreover, the gravity of irAE can be also exacerbated by a combination of ICI treatments. A recent report has demonstrated that irAEs could affect only 16.3% and 27.3% of patients treated with Nivolumab or Ipilimumab, respectively. In contrast, 55.0% of the patients co-treated with Ipilimumab and Nivolumab suffered from high-level toxicity, thus resulting in the discontinuation of the therapy in more than one third of the cases [112]. Therefore, irAEs can be therapeutically detrimental with an increasing trend of combo therapies, considering that the related toxicity needs to be controlled without impairing the ideal anti-tumor effect of ICIs. Still, the tolerance, overall safety, and rate of discontinuation in patients treated with ICIs are generally more positive than for those receiving chemotherapy [113]. To increase the beneficial effects of immunotherapy, careful attention must be paid to avoid irAEs as much as possible from the initial developmental process of ICI treatment, particularly considering challenging characteristics of GBM. It is also crucial to concurrently develop techniques for the early detection of irAEs and interventional strategies to control their severity.

## 7. Current Challenges of Glioblastoma Treatment with Immune-Checkpoint Inhibitors

To maximize the therapeutic effect of ICI-based treatments in GBM patients, numerous challenges should be listed and addressed. For this work, it is critical to have an in-depth understanding of all factors that might influence the ICI therapeutic outcomes. Similarly, understanding the underlying mechanisms of how certain GBM patients show a complete and long-lasting response, while others suffer from tumor relapse in the first few months of therapy is also important [114,115]. In some cases, the adverse response to the ICI treatment is related with the fact that many advanced GBM patients undergo extensive pre-treatment with several traditional therapies. As a consequence, when these patients receive checkpoint inhibitors, not only invasive and immunosuppressive characteristics of GBM but also the sustained interference of the patient immunity due to pre-treatment may reduce the meaningful response to the ICI treatment. Another basic limitation to ICI-based treatment is that specific ICIs may require pre-existing anti-tumor immunity of patients for the curative effect. In fact, a representative number of patients have no natural immune response to GBM. In the context of CTLA-4 blocking, current data is quite conflicting. One particular study has shown that a pre-existing immunologically active TME is correlated with favored therapeutic response to Ipilimumab [116]. In contrast, data from other studies have indicated that CTLA-4 blocking improves the reservoir of tumor-specific T cells, which are not detected before treatment. Therefore, to overcome the limited immune response of naïve patients against tumors, strategies to stimulate tumor-specific T-cell response such as DC-based cancer vaccines should be considered as precursors to immune checkpoint strategy. Indeed, these strategies may increase the rate of patients who could benefit from ICI-based therapy. 

The use of immunotherapy to stimulate host immune system towards a systemic anti-tumor immune response might become a promising direction for GBM therapeutics. In fact, this approach allows sustainable and dramatic immune responses in different types of tumors and now is a key focus of GBM. However, systemic response and long-lasting OS by immunotherapy in GBM remains unmet possibly due to multifactorial reasons/obstacles including (1) a highly immunosuppressive milieu of GBM and TME; (2) the great heterogeneity and relatively low TMB; (3) the low permeability and high CNS constraints of immune therapeutic drugs across the BBB; (4) the deficiency of immunogenic tumor antigens; (5) a profoundly immunosuppressive standard care for GBM such as the use of corticosteroids; (6) the limited infiltration of T cells related with suppression of systemic immunity; (7) the favored focus on T cells and the neglection of other immune cells, particularly myeloid cells. Such factors counteract the immune stimulatory efficiency of ICIs and thereby may confound the findings in GBM treatment compared to other more immunogenic tumors [117,118,119,120,121,122,123,124]. 

## 8. Conclusions

Despite some challenges in the field of ICI treatment, the success of the therapeutic strategies towards various cancers in the past ten years has highlighted the significant role of the immune system in tumor control. Moreover, historical findings based on numerous clinical studies have clearly guided the potential effects of ICIs treatment, thus injecting new vitality into the field of cancer therapy. Clinically, ICI-based treatments have been applied to the limited targets of PD-1, PD-L1 and CTLA4 stimulating adaptive immunity so far. However, many other immune checkpoint approaches involving both adaptive and innate immunity have been recently discovered, which could later serve as probable targets for monotherapy or combinational ICI therapy. 

GBM is a complex tumor that involves different and complicated molecular pathways and TMEs. Although a great deal of investigations have been pursued, the current achievements towards GBM treatments are still insufficient. Particularly, clinical outcome of GBM has been hindered by its unique cancer-associated immune suppression and immune escape. There have been recent advances in the treatment of GBM leveraging a variety of platforms for cancer immunotherapy to overcome immune suppression. Nevertheless, some initial results of clinical trials have been disappointing with only a small subset of patients that responded to single blocking agent. Thus, combining immune-checkpoint agents with different functional mechanisms to improve the OS of GBM patients is highly desirable to address this unmet clinical need. Novel immune checkpoint inhibitors targeting myeloid cells, nanomaterial-based enhanced drug delivery strategy, and careful design of treatment regimens to improve T-cell functionality also provide new potential to improve immunotherapy outcome for GBM. Ultimately, we hope that vigorous ongoing and future studies may better guide the way in which immunotherapy can be applied in the clinics as part of the standard of care for patients suffering from GBM.

## Figures and Tables

**Table 1:** Recent clinical studies with immune-checkpoint inhibitors and some combinational therapies targeting glioblastoma.

Therapeutic Approach	Immune Targets	Type of Glioma	Type of Study	Number of Subjects	Overall Survival (OS)	Progression Free Survival (PFS)	Clinical Trial Identifier	Ref No.
**Immune-Checkpoint Inhibitors and Combinational Therapies**								
Nivolumab vs. Bevacizumab	PD-1	Recurrent glioblastoma	Phase III	369	9.8 vs. 10.0 months	1.5 vs. 3.5 months	NCT02017717	[44]
Neoadjuvant Nivolumab	PD-1	Primary and recurrent glioblastoma	Phase II	30	7.3 months	4.1 months	NCT02550249	[45]
Nivolumab + Radiation + Bevacizumab	PD-1	Recurrent glioblastoma	Phase II	94 (recruiting)	N/A	N/A	NCT03743662	[46]
Pembrolizumab	PD-1	Refractory high grade glioma	Retrospective study	25	4 months	1.4 months	N/A	[47]
Nivolumab + Temozolomide	PD-1	Glioblastoma	Phase II	102 (recruiting)	N/A	N/A	NCT04195139	[48]
CAR T Cells ± Nivolumab and Ipilimumab	PD-1, IL13Ralpha2, CTLA4	Recurrent or refractory glioblastoma	Phase I	60 (recruiting)	N/A	N/A	NCT04003649	[49]
Ipilimumab + Nivolumab	PD-1 and CTLA4	Glioblastoma	Phase I	6	N/A	N/A	NCT03233152	[50]
Ipilimumab + Nivolumab	PD-1 and CTLA4	Recurrent and secondary glioblastoma	Phase II	37 (not yet recruiting)	N/A	N/A	NCT04145115	[51]
DC vaccines + Nivolumab	CTLA 4	Recurrent glioblastoma	Phase I	6	15.3 months with surgery	6.3 months with surgery	NCT02529072	[52]
Oncolytic adenovirus (DNX-2401) + Pembrolizumab	PD-L1	Recurrent glioblastoma or gliosarcoma	Phase II	49 (not yet recruiting)	N/A	N/A	NCT02798406	[53]
Stereotactic radiosurgery (SRS) + Spartalizumab + MBG453	TIM3 + PD-1 + SRS	Recurrent glioblastoma	Phase I	15 (recruiting)	N/A	N/A	NCT03961971	[54]
Nivolumab + BMS-986205 + Radiotherapy + Temozolomide	IDO + PD-1	Glioblastoma	Phase I	30 (recruiting)	N/A	N/A	NCT04047706	[55]
Indoximod + Radiation + Temozolomide	IDO	Pediatric glioblastoma	Phase I	29	N/A	6.2 months	NCT02502708	[56]
**Oncolytic Viruses**								
Oncolytic virus TG6002 + 5-flucytosine	N/A	Recurrent glioblastoma	Phase I/Phase II	78 (recruiting)	N/A	N/A	NCT03294486	[57]
Engineered herpes virus G207	N/A	Recurrent glioblastoma	Phase Ib	6	6.6 months	N/A	NCT00028158	[58]
DNX-2401 + Interferon gamma (IFN-γ)	N/A	Recurrent glioblastoma	Phase Ib	27	N/A	N/A	NCT02197169	[59]
Polio/Rhinovirus recombinant (PVSRIPO)	N/A	Recurrent glioblastoma	Phase Ib	61	12.5 months	N/A	NCT01491893	[60,61]
**DC Vaccines**								
Pembrolizumab + DC vaccine (ATL-DC)	N/A	Recurrent glioblastoma	Phase I	40 (recruiting)	N/A	N/A	NCT04201873	[62]
Pp65-DCs + Temozolomide	N/A	Glioblastoma	Phase II	100 (ongoing)	N/A	N/A	NCT02366728	[63]
**CAR T Therapy**								
Anti-EGFRvIII CAR T cells + Cyclophosphamide	EGFRvIII	Recurrent glioblastoma	Pilot trial	20 (estimated)	N/A	N/A	NCT02844062	[64]
IL13Ralpha2-specific CAR	IL13Ra2	Recurrent glioblastoma or refractory high grade glioma	Pilot trial	3	11 months (mean survival)	N/A	NCT00730613	[65]
HER2-CAR VSTs	HER2	Progressive HER2+ glioblastoma	Phase I	17	11.1 months	N/A	NCT01109095	[66]

PD-1, Programmed cell death 1; PD-L1, Programmed cell death ligand 1; CTLA-4, Cytotoxic T lymphocyte protein 4; IDO, Idolamine 2, 3-dioxygenase; CAR T Cells, Chimeric antigen receptors T cells; SRS, Stereotactic radiosurgery; DC vaccines, Dendritic cell vaccines; IFN-γ, Interferon gamma. PVSRIPO, Polio/Rhinovirus Recombinant; ATL-DC, Autologous tumor lysate-pulsed dendritic cell vaccine; Pp65-DCs, Pp65-dendritic cells; Anti-EGFRvIII CAR, anti-epidermal growth factor receptor variant III chimeric antigen receptors; IL13Ralpha2-specific CAR, Interleukin-13 receptor subunit alpha2-specific chimeric antigen receptors. 5-FC, 5-flucytosine; HER2-CAR VSTs, Human epidermal growth factor receptor 2- chimeric antigen receptors virus-specific T cells. OS and PFS were depicted from the time of inoculation. Data taken from https://clinicaltrials.gov/.

**Table 2:** Management of immune-related adverse events induced by immune-checkpoint inhibitors.

irAE	ICIs Treatment	Management
Inflammatory Arthritis	anti-PD-1 and CTLA4 (Ipilimumab/Nivolumab)	Acetaminophen + oral corticosteroids of prednisone + intra-articular corticosteroid injection
		ICIs resumed
Temporal Arteritis	anti-PD-L1 (Durvalumab)	Prednisone
		ICIs resumed pending clinical response
Myocarditis	anti-PD-1 and CTLA4 (Ipilimumab + Nivolumab)	Methylprednisolone and diuretic treatment
		ICIs permanently discontinued
Maculopapular Rash	anti-PD-L1 (Avelumab)	Betamethasone treatment+oral prednisone
		Resumed after symptom relief below grade 1 toxicity
Nephritis	anti-PD-1 (Pembrolizumab)	Corticosteroids
		ICIs resumed after renal indices in a normal range
Encephalitis	anti-PD-1 and CTLA4 (Ipilimumab + Nivolumab)	Pulse corticosteroids
		Resumed after neurological recovery
Myasthenia Gravis	anti-PD-1 (Nivolumab)	Corticosteroids
		Permanently discontinued
Uveitus	anti-PD-L1 (Atezolizumab)	Topical cycloplegic agent + prednisone
		Continued until corticosteroid reduction completed
Pneumonitis	anti-PD-L1 (Durvalumab)	Dose-dependent corticosteroids according to different grade of checkpoint-inhibitor pneumonitis (CIP)
		Temporarily held during corticosteroid treatment in grade I and 2 CIP toxicity
Hypophysitis	anti-PD-1 and CTLA4 (Ipilimumab + Nivolumab)	hydrocortisone +levothyroxine
		Resumed
Hypothyroidism	anti-PD-L1 (Durvalumab)	levothyroxine
		Continued
Thrombocytopenia	anti-PD-1 (Pembrolizumab)	Prednisone
		Resumed after 4 weeks treatment
Colitis	anti-PD-1 and CTLA4 (Ipilimumab + Nivolumab)	Prednisone
		Resumed

irAE, Immune-related adverse event; ICIs, Immune checkpoint inhibitors; anti-PD-1, anti-programmed cell death 1; CTLA4, Cytotoxic T lymphocyte protein 4; anti-PD-L1, anti-programmed cell death ligand 1; CIP, Checkpoint-inhibitor pneumonitis.

## References

[1] Stupp R., Hegi M.E., Mason W.P., van den Bent M.J., Taphoorn M., Janzer R.C., Ludwin S.K., Allgeier A., Fisher B., Belanger K. (2009). Effects of radiotherapy with concomitant and adjuvant temozolomide versus radiotherapy alone on survival in glioblastoma in a randomised phase III study: 5-Year analysis of the EORTC-NCIC trial. Lancet Oncol..

[2] Stupp R., Mason W.P., van den Bent M.J., Weller M., Fisher B., Taphoorn M.J.B., Belanger K., Brandes A.A., Marosi C., Bogdahn U. (2005). Radiotherapy plus Concomitant and Adjuvant Temozolomide for Glioblastoma. N. Engl. J. Med..

[3] Ostrom Q.T., Gittleman H., Fulop J., Liu M., Blanda R., Kromer C., Wolinsky Y., Kruchko C., Barnholtz-Sloan J.S. (2015). CBTRUS statistical report: Primary brain and central nervous system tumors diagnosed in the united states in 2008-2012. Neuro-Oncology.

[4] Davies D.C. (2002). Blood–brain barrier breakdown in septic encephalopathy and brain tumours. J. Anat..

[5] Schlageter K.E., Molnar P., Lapin G.D., Groothuis D.R. (1999). Microvessel organization and structure in experimental brain tumors: Microvessel populations with distinctive structural and functional properties. Microvasc. Res..

[6] Lombardi G., Rumiato E., Bertorelle R., Saggioro D., Farina P., Della Puppa A., Zustovich F., Berti F., Sacchetto V., Marcato R. (2015). Clinical and Genetic Factors Associated with Severe Hematological Toxicity in Glioblastoma Patients during Radiation plus Temozolomide Treatment: A prospective study. Am. J. Clin. Oncol..

[7] Grossman S.A., Ye X.B., Lesser G., Sloan A., Carraway H., Desideri S., Piantadosi S. (2011). Immunosuppression in patients with High-Grade gliomas treated with radiation and temozolomide. Clin. Cancer Res..

[8] Stummer W., Pichlmeier U., Meinel T., Wiestler O.D., Zanella F., Reulen H. (2006). Fluorescence-guided surgery with 5-aminolevulinic acid for resection of malignant glioma: A randomised controlled multicentre phase III trial. Lancet Oncol..

[9] McGirt M.J., Chaichana K.L., Attenello F.J., Weingart J.D., Than K., Burger P.C., Olivi A., Brem H., Quinoñes-Hinojosa A. (2008). Extent of surgical resection is independently associated with survival in patients with hemispheric infiltrating low-grade gliomas. Neurosurgery.

[10] Weller M., Cloughesy T., Perry J.R., Wick W. (2013). Standards of care for treatment of recurrent glioblastoma—Are we there yet?. Neuro-oncology.

[11] Gramatzki D., Dehler S., Rushing E.J., Zaugg K., Hofer S., Yonekawa Y., Bertalanffy H., Valavanis A., Korol D., Rohrmann S. (2016). Glioblastoma in the Canton of Zurich, Switzerland revisited: 2005 to 2009. Cancer.

[12] Gallego O. (2015). Nonsurgical treatment of recurrent glioblastoma. Curr. Oncol..

[13] Billingham R.E., Brent L., Medawar P.B. (1953). ’Actively acquired tolerance’ of foreign cells. Nature.

[14] Billingham R.E., Brent L., Medawar P.B., Sparrow E.M. (1954). Quantitative studies on tissue transplantation immunity. I. The survival times of skin homografts exchanged between members of different inbred strains of mice. Proc. R. Soc. Lond. B Biol. Sci..

[15] Medawar P.B. (1948). Immunity to homologous grafted skin. III. The fate of skin homographs transplanted to the brain, to subcutaneous tissue, and to the anterior chamber of the eye. Br. J. Exp. Pathol..

[16] Lim M., Xia Y.X., Bettegowda C., Weller M. (2018). Current state of immunotherapy for glioblastoma. Nat. Rev. Clin. Oncol..

[17] Louveau A., Smirnov I., Keyes T.J., Eccles J.D., Rouhani S.J., Peske J.D., Derecki N.C., Castle D., Mandell J.W., Lee K.S. (2015). Structural and functional features of central nervous system lymphatic vessels. Nature.

[18] Kipnis J. (2016). Multifaceted Interactions Between Adaptive Immunity and the Central Nervous System. Science.

[19] Bloch O., Crane C.A., Kaur R., Safaee M., Rutkowski M.J., Parsa A.T. (2013). Gliomas promote immunosuppression through induction of B7-H1 expression in tumor-associated macrophages. Clin. Cancer Res..

[20] Wainwright D.A., Chang A.L., Dey M., Balyasnikova I.V., Kim C.K., Tobias A., Cheng Y., Kim J.W., Qiao J., Zhang L. (2014). Durable therapeutic efficacy utilizing combinatorial blockade against IDO, CTLA-4 and PD-L1 in mice with brain tumors. Clin. Cancer Res..

[21] McGranahan T., Li G., Nagpal S. (2017). History and current state of immunotherapy in glioma and brain metastasis. Ther. Adv. Med. Oncol..

[22] Sampson J.H., Maus M.V., June C.H. (2017). Immunotherapy for brain tumors. J. Clin. Oncol..

[23] Hodges T.R., Ott M., Xiu J., Gatalica Z., Swensen J., Zhou S., Huse J.T., de Groot J., Li S.L., Overwijk W.W. (2017). Mutational burden, immune checkpoint expression, and mismatch repair in glioma: Implications for immune checkpoint immunotherapy. Neuro-Oncology.

[24] Bouffet E., Larouche V., Campbell B.B., Merico D., de Borja R., Aronson M., Durno C., Krueger J., Cabric V., Ramaswamy V. (2016). Immune checkpoint inhibition for hypermutant glioblastoma multiforme resulting from germline biallelic mismatch repair deficiency. J. Clin. Oncol..

[25] Johanns T.M., Miller C.A., Dorward I.G., Tsien C., Chang E., Perry A., Uppaluri R., Ferguson C., Schmidt R.E., Dahiya S. (2016). Immunogenomics of hypermutated glioblastoma: A patient with germline POLE deficiency treated with checkpoint blockade immunotherapy. Cancer Discov..

[26] Feng M., Jiang W., Kim B.Y.S., Zhang C.C., Fu Y.X., Weissman I.L. (2019). Phagocytosis Checkpoints as New Targets for Cancer Immunotherapy. Nat Rev Cancer..

[27] Zhao P.F., Wang Y.H., Kang X.J., Wu A.H., Yin W.M., Tang Y.S., Wang J.Y., Zhang M., Duan Y.F., Huang Y.Z. (2018). Dual-targeting biomimetic delivery for anti-glioma activity via remodeling the tumor microenvironment and directing macrophage-mediated immunotherapy. Chem. Sci..

[28] Li Q.Y., Barres B.A. (2018). Microglia and macrophages in brain homeostasis and disease. Nat. Rev. Immunol..

[29] Ginhoux F., Greter M., Leboeuf M., Nandi S., See P., Gokhan S., Mehler M.F., Conway S.J., Ng L.G., Stanley E.R. (2010). Fate mapping analysis reveals that adult microglia derive from primitive macrophages. Science.

[30] Ajami B., Bennett J.L., Krieger C., McNagny K.M., Rossi F.M.V. (2011). Infiltrating monocytes trigger EAE progression, but do not contribute to the resident microglia pool. Nat. Neurosci..

[31] Bennett F.C., Bennett M.L., Yaqoob F., Mulinyawe S.B., Grant G.A., Hayden Gephart M., Plowey E.D., Barres B.A. (2018). A combination of ontogeny and CNS environment establishes microglial identity. Neuron.

[32] Graeber M.B., Scheithauer B.W., Kreutzberg G.W. (2002). Microglia in brain tumors. Glia.

[33] Matias D., Balça-Silva J., Da Graça G.C., Wanjiru C.M., Macharia L.W., Nascimento C.P., Roque N.R., Coelho-Aguiar J.M., Pereira C.M., Dos Santos M.F. (2018). Microglia/Astrocytes–glioblastoma crosstalk: Crucial molecular mechanisms and microenvironmental factors. Front. Cell. Neurosci..

[34] Hussein M.R. (2006). Tumour-associated macrophages and melanoma tumourigenesis: Integrating the complexity. Int. J. Exp. Pathol..

[35] Aldape K., Brindle K.M., Chesler L., Chopra R., Gajjar A., Gilbert M.R., Gottardo N., Gutmann D.H., Hargrave D., Holland E.C. (2019). Challenges to curing primary brain tumours. Nat. Rev. Clin. Oncol..

[36] Pyonteck S.M., Akkari L., Schuhmacher A.J., Bowman R.L., Sevenich L., Quail D.F., Olson O.C., Quick M.L., Huse J.T., Teijeiro V. (2013). CSF-1R inhibition alters macrophage polarization and blocks glioma progression. Nat. Med..

[37] Zeiner P.S., Preusse C., Blank A.E., Zachskorn C., Baumgarten P., Caspary L., Braczynski A.K., Weissenberger J., Bratzke H., Reiß S. (2015). MIF receptor CD74 is restricted to Microglia/Macrophages, associated with a m1-polarized immune milieu and prolonged patient survival in gliomas. Brain Pathol..

[38] Pong W.W., Walker J., Wylie T., Magrini V., Luo J., Emnett R.J., Choi J., Cooper M.L., Griffith M., Griffith O.L. (2013). F11R is a novel monocyte prognostic biomarker for malignant glioma. PLoS ONE.

[39] Hambardzumyan D., Gutmann D.H., Kettenmann H. (2016). The role of microglia and macrophages in glioma maintenance and progression. Nat. Neurosci..

[40] Mantovani A., Marchesi F., Malesci A., Laghi L., Allavena P. (2017). Tumour-associated macrophages as treatment targets in oncology. Nat. Rev. Clin. Oncol..

[41] Arlauckas S.P., Garris C.S., Kohler R.H., Kitaoka M., Cuccarese M.F., Yang K.S., Miller M.A., Carlson J.C., Freeman G.J., Anthony R.M. (2017). In vivo imaging reveals a tumor-associated macrophage-mediated resistance pathway in anti-PD-1 therapy. Sci. Transl. Med..

[42] Wang H.X., Xu T., Huang Q.L., Jin W.L., Chen J.X. (2020). Immunotherapy for malignant glioma: Current status and future directions. Trends Pharmacol. Sci..

[43] Tarhini A.A., Iqbal F. (2010). CTLA-4 blockade: Therapeutic potential in cancer treatments. Oncotargets Ther..

[44] ClinicalTrials.gov (2013). A Study of the Effectiveness and Safety of Nivolumab Compared to Bevacizumab and of Nivolumab with or without Ipilimumab in Glioblastoma Patients (CheckMate 143). https://clinicaltrials.gov/show/NCT02017717.

[45] ClinicalTrials.gov (2015). Neoadjuvant Nivolumab in Glioblastoma (Neo-Nivo). https://clinicaltrials.gov/show/NCT02550249.

[46] ClinicalTrials.gov (2018). Nivolumab with Radiation Therapy and Bevacizumab for Recurrent MGMT Methylated Glioblastoma. https://clinicaltrials.gov/show/NCT03743662.

[47] Reiss S.N., Yerram P., Modelevsky L., Grommes C. (2017). Retrospective review of safety and efficacy of programmed cell death-1 inhibitors in refractory high grade gliomas. J. Immunother. Cancer.

[48] ClinicalTrials.gov (2018). Nivolumab and Temozolomide versus Temozolomide Alone in Newly Diagnosed Elderly Patients with GBM (NUTMEG). https://clinicaltrials.gov/show/NCT04195139.

[49] ClinicalTrials.gov (2019). IL13Ralpha2-Targeted Chimeric Antigen Receptor (CAR) t Cells with or without Nivolumab and Ipilimumab in Treating Patients with Recurrent or Refractory Glioblastoma. https://clinicaltrials.gov/show/NCT04003649.

[50] ClinicalTrials.gov (2016). Intra-Tumoral Ipilimumab Plus Intravenous Nivolumab Following the Resection of Recurrent Glioblastoma (GlitIpNi). https://clinicaltrials.gov/show/NCT03233152.

[51] ClinicalTrials.gov (2020). A Study Testing the Effect of Immunotherapy (Ipilimumab and Nivolumab) in Patients with Recurrent Glioblastoma with Elevated Mutational Burden. https://clinicaltrials.gov/show/NCT04145115.

[52] ClinicalTrials.gov (2016). Nivolumab with DC Vaccines for Recurrent Brain Tumors (AVERT). https://clinicaltrials.gov/show/NCT02529072.

[53] ClinicalTrials.gov (2016). Combination Adenovirus + Pembrolizumab to Trigger Immune Virus Effects (CAPTIVE). https://clinicaltrials.gov/show/NCT02798406.

[54] ClinicalTrials.gov (2020). Trial of Anti-Tim-3 in Combination with Anti-PD-1 and SRS in Recurrent GBM. https://clinicaltrials.gov/show/NCT03961971.

[55] ClinicalTrials.gov (2019). Nivolumab, BMS-986205, and Radiation Therapy with or without Temozolomide in Treating Patients with Newly Diagnosed Glioblastoma. https://clinicaltrials.gov/show/NCT04047706.

[56] ClinicalTrials.gov (2015). Study of the IDO Pathway Inhibitor, Indoximod, and Temozolomide for Pediatric Patients with Progressive Primary Malignant Brain Tumors. https://clinicaltrials.gov/show/NCT02502708.

[57] ClinicalTrials.gov (2017). Safety and Efficacy of the ONCOlytic VIRus Armed for Local Chemotherapy, TG6002/5-FC, in Recurrent Glioblastoma Patients (ONCOVIRAC). https://clinicaltrials.gov/show/NCT03294486.

[58] ClinicalTrials.gov (2017). Safety and Effectiveness Study of g207, a Tumor-Killing Virus, in Patients with Recurrent Brain Cancer. https://clinicaltrials.gov/show/NCT00028158.

[59] ClinicalTrials.gov (2014). DNX-2401 with Interferon Gamma (IFN-γ) for Recurrent Glioblastoma or Gliosarcoma Brain Tumors (TARGET-I). https://clinicaltrials.gov/show/NCT02197169.

[60] ClinicalTrials.gov (2012). PVSRIPO for Recurrent Glioblastoma (GBM) (PVSRIPO). https://clinicaltrials.gov/show/NCT01491893.

[61] Desjardins A., Gromeier M., Herndon J.E., Beaubier N., Bolognesi D.P., Friedman A.H., Friedman H.S., McSherry F., Muscat A.M., Nair S. (2018). Recurrent Glioblastoma Treated with Recombinant Poliovirus. N. Engl. J. Med..

[62] ClinicalTrials.gov (2020). Pembrolizumab and a Vaccine (ATL-DC) for the Treatment of Surgically Accessible Recurrent Glioblastoma. https://clinicaltrials.gov/show/NCT04201873.

[63] ClinicalTrials.gov (2015). DC Migration Study for Newly-Diagnosed GBM (ELEVATE). https://clinicaltrials.gov/show/NCT02366728.

[64] ClinicalTrials.gov (2016). Pilot Study of Autologous Anti-EGFRvIII CAR t cells in Recurrent Glioblastoma Multiforme. https://clinicaltrials.gov/show/NCT02844062.

[65] ClinicalTrials.gov (2002). Cellular Adoptive Immunotherapy Using Genetically Modified T-Lymphocytes in Treating Patients with Recurrent or Refractory High-Grade Malignant Glioma. https://clinicaltrials.gov/show/NCT00730613.

[66] ClinicalTrials.gov (2010). CMV-Specific Cytotoxic t Lymphocytes Expressing CAR Targeting HER2 in Patients with GBM (HERT-GBM). https://clinicaltrials.gov/show/NCT01109095.

[67] Luke J.J., Ott P.A. (2015). PD-1 pathway inhibitors: The next generation of immunotherapy for advanced melanoma. Oncotarget.

[68] Darvin P., Toor S.M., Sasidharan Nair V., Elkord E. (2018). Immune Checkpoint Inhibitors: Recent Progress and Potential Biomarkers. Exp. Mol. Med..

[69] Wei S.C., Duffy C.R., Allison J.P. (2018). Fundamental Mechanisms of Immune Checkpoint Blockade Therapy. Cancer Discov..

[70] Romani M., Pistillo M.P., Carosio R., Morabito A., Banelli B. (2018). Immune checkpoints and innovative therapies in glioblastoma. Front. Oncol..

[71] Cheng X.X., Veverka V., Radhakrishnan A., Waters L.C., Muskett F.W., Morgan S.H., Huo J., Yu C., Evans E.J., Leslie A.J. (2013). Structure and interactions of the human programmed cell death 1 receptor. J. Biol. Chem..

[72] Francisco L.M., Salinas V.H., Brown K.E., Vanguri V.K., Freeman G.J., Kuchroo V.K., Sharpe A.H. (2009). PD-L1 regulates the development, maintenance, and function of induced regulatory T cells. J. Exp. Med..

[73] Nduom E.K., Wei J., Yaghi N.K., Huang N., Kong L., Gabrusiewicz K., Ling X.Y., Zhou S.H., Ivan C., Chen J.Q. (2016). PD-L1 expression and prognostic impact in glioblastoma. Neuro-Oncology.

[74] Berghoff A.S., Kiesel B., Widhalm G., Rajky O., Ricken G., Wöhrer A., Dieckmann K., Filipits M., Brandstetter A., Weller M. (2015). Programmed death ligand 1 expression and tumor-infiltrating lymphocytes in glioblastoma. Neuro-Oncology.

[75] Omuro A., Vlahovic G., Lim M., Sahebjam S., Baehring J., Cloughesy T., Voloschin A., Ramkissoon S.H., Ligon K.L., Latek R. (2018). Nivolumab with or without ipilimumab in patients with recurrent glioblastoma: Results from exploratory phase I cohorts of CheckMate 143. Neuro-Oncology.

[76] Ribas A., Wolchok J.D. (2018). Cancer immunotherapy using checkpoint blockade. Science.

[77] Gholamin S., Mitra S.S., Feroze A.H., Liu J., Kahn S.A., Zhang M., Esparza R., Richard C., Ramaswamy V., Remke M. (2017). Disrupting the CD47-SIRPα anti-phagocytic axis by a humanized anti-CD47 antibody is an efficacious treatment for malignant pediatric brain tumors. Sci. Transl. Med..

[78] Willingham S.B., Volkmer J., Gentles A.J., Sahoo D., Dalerba P., Mitra S.S., Wang J., Contreras-Trujillo H., Martin R., Cohen J.D. (2012). The CD47-signal regulatory protein alpha (SIRPa) interaction is a therapeutic target for human solid tumors. Proc. Natl. Acad. Sci. USA.

[79] Edris B., Weiskopf K., Volkmer A.K., Volkmer J., Willingham S.B., Contreras-Trujillo H., Liu J., Majeti R., West R.B., Fletcher J.A. (2012). Antibody therapy targeting the CD47 protein is effective in a model of aggressive metastatic leiomyosarcoma. Proc. Natl. Acad. Sci. USA.

[80] Zhang X.Y., Fan J.J., Wang S.F., Li Y.B., Wang Y.C., Li S., Luan J.Y., Wang Z.Y., Song P., Chen Q.C. (2017). Targeting CD47 and autophagy elicited enhanced antitumor effects in Non-Small cell lung cancer. Cancer Immunol. Res..

[81] Zhu H.Y., Leiss L.N., Yang N., Rygh C.B., Mitra S.S., Cheshier S.H., Weissman I.L., Huang B., Miletic H., Bjerkvig R. (2017). Surgical debulking promotes recruitment of macrophages and triggers glioblastoma phagocytosis in combination with CD47 blocking immunotherapy. Oncotarget.

[82] Sockolosky J.T., Dougan M., Ingram J.R., Chia C.M.H., Kauke M.J., Almo S.C., Ploegh H.L., Garcia K.C. (2016). Durable antitumor responses to CD47 blockade require adaptive immune stimulation. Proc. Natl. Acad. Sci. USA.

[83] Zhang M., Hutter G., Kahn S.A., Azad T.D., Gholamin S., Xu C.Y., Liu J., Achrol A.S., Richard C., Sommerkamp P. (2016). Anti-CD47 treatment stimulates phagocytosis of glioblastoma by m1 and m2 polarized macrophages and promotes m1 polarized macrophages in vivo. PLoS ONE.

[84] Li F., Lv B.K., Liu Y., Hua T., Han J.B., Sun C.M., Xu L.M., Zhang Z.F., Feng Z.M., Cai Y.Q. (2018). Blocking the CD47-SIRPalpha axis by delivery of anti-CD47 antibody induces antitumor effects in glioma and glioma stem cells. Oncoimmunology.

[85] Das M., Zhu C., Kuchroo V.K. (2017). Tim-3 and its role in regulating anti-tumor immunity. Immunol. Rev..

[86] Han S., Feng S.Z., Xu L.S., Shi W.W., Wang X.H., Wang H., Yu C.Y., Dong T., Xu M.H., Liang G.B. (2014). Tim-3 on peripheral CD4+ and CD8+ t cells is involved in the development of glioma. DNA Cell Biol..

[87] Li G.Z., Wang Z., Zhang C.B., Liu X., Cai J.Q., Wang Z.L., Hu H.M., Wu F., Bao Z.S., Liu Y.W. (2017). Molecular and clinical characterization of TIM-3 in glioma through 1,024 samples. Oncoimmunology.

[88] Prendergast G.C., Smith C., Thomas S., Mandik-Nayak L., Laury-Kleintop L., Metz R., Muller A.J. (2014). Indoleamine 2, 3-dioxygenase pathways of pathogenic inflammation and immune escape in cancer. Cancer Immunol. Immunother..

[89] Cheong J.E., Ekkati A., Sun L.J. (2018). A patent review of IDO1 inhibitors for cancer. Expert Opin. Ther. Pat..

[90] Hanihara M., Kawataki T., Oh-Oka K., Mitsuka K., Nakao A., Kinouchi H. (2016). Synergistic antitumor effect with indoleamine 2, 3-dioxygenase inhibition and temozolomide in a murine glioma model. J. Neurosurg..

[91] Koyama S., Akbay E.A., Li Y.Y., Herter-Sprie G.S., Buczkowski K.A., Richards W.G., Gandhi L., Redig A.J., Rodig S.J., Asahina H. (2016). Adaptive resistance to therapeutic PD-1 blockade is associated with upregulation of alternative immune checkpoints. Nat. Commun..

[92] Kim J.E., Patel M.A., Mangraviti A., Kim E.S., Theodros D., Velarde E., Liu A., Sankey E.W., Tam A., Xu H.Y. (2017). Combination therapy with Anti-PD-1, Anti-TIM-3, and focal radiation results in regression of murine gliomas. Clin. Cancer Res..

[93] Sun F., Guo Z.S., Gregory A.D., Shapiro S.D., Xiao G., Qu Z. (2019). Dual but not single PD-1 or TIM-3 blockade enhances oncolytic virotherapy in refractory lung cancer. J Immunother. Cancer..

[94] Patel M.A., Kim J.E., Theodros D., Tam A., Velarde E., Kochel C.M., Francica B., Nirschl T.R., Ghasemzadeh A., Mathios D. (2016). Agonist anti-GITR monoclonal antibody and stereotactic radiation induce immune-mediated survival advantage in murine intracranial glioma. J. Immunother. Cancer.

[95] Sharp M., Corp D., Washington University School of Medicine MK-3475 in Combination with MRI-Guided Laser Ablation in Recurrent Malignant Gliomas. https://clinicaltrials.gov/ct2/show/NCT02311582.

[96] Hitchcock S.A. (2008). Blood–brain barrier permeability considerations for CNS-targeted compound library design. Curr. Opin. Chem. Biol..

[97] Pardridge W.M. (2007). Blood–brain barrier delivery. Drug Discov. Today.

[98] Weiss N., Miller F., Cazaubon S., Couraud P. (2009). The blood-brain barrier in brain homeostasis and neurological diseases. Biochim. Biophys. Acta (BBA)-Biomembr..

[99] Da Ros M., De Gregorio V., Iorio A.L., Giunti L., Guidi M., De Martino M., Genitori L., Sardi I. (2018). Glioblastoma chemoresistance: The double play by microenvironment and blood-brain barrier. Int. J. Mol. Sci..

[100] Liu Y.Y., Ran R., Chen J.T., Kuang Q.F., Tang J., Mei L., Zhang Q.Y., Gao H.L., Zhang Z.R., He Q. (2014). Paclitaxel loaded liposomes decorated with a multifunctional tandem peptide for glioma targeting. Biomaterials.

[101] Shi J.L., Hou S.Q., Huang J.F., Wang S.S., Huan W., Huang C.J., Liu X.J., Jiang R., Qian W.B., Lu J.J. (2017). An MSN-PEG-IP drug delivery system and IL13Rα\alphaα2 as targeted therapy for glioma. Nanoscale.

[102] Strauss L., Mahmoud M.A.A., Weaver J.D., Tijaro-Ovalle N.M., Christofides A., Wang Q., Pal R., Yuan M., Asara J., Patsoukis N. (2020). Targeted deletion of PD-1 in myeloid cells induces antitumor immunity. Sci. Immunol..

[103] Goswami S., Walle T., Cornish A.E., Basu S., Anandhan S., Fernandez I., Vence L., Blando J., Zhao H., Yadav S.S. (2020). Immune profiling of human tumors identifies CD73 as a combinatorial target in glioblastoma. Nat. Med..

[104] Filley A.C., Henriquez M., Dey M. (2017). Recurrent glioma clinical trial, CheckMate-143: The game is not over yet. Oncotarget.

[105] Cloughesy T.F., Mochizuki A.Y., Orpilla J.R., Hugo W., Lee A.H., Davidson T.B., Wang A.C., Ellingson B.M., Rytlewski J.A., Sanders C.M. (2019). Neoadjuvant anti-PD-1 immunotherapy promotes a survival benefit with intratumoral and systemic immune responses in recurrent glioblastoma. Nat. Med..

[106] Forde P.M., Chaft J.E., Smith K.N., Anagnostou V., Cottrell T.R., Hellmann M.D., Zahurak M., Yang S.C., Jones D.R., Broderick S. (2018). Neoadjuvant PD-1 blockade in resectable lung cancer. N. Engl. J. Med..

[107] Schalper K.A., Rodriguez-Ruiz M.E., Diez-Valle R., López-Janeiro A., Porciuncula A., Idoate M.A., Inogés S., de Andrea C., López-Diaz De Cerio A., Tejada S. (2019). Neoadjuvant nivolumab modifies the tumor immune microenvironment in resectable glioblastoma. Nat. Med..

[108] El Osta B., Hu F., Sadek R., Chintalapally R., Tang S.C. (2017). Not all immune-checkpoint inhibitors are created equal: Meta-analysis and systematic review of immune-related adverse events in cancer trials. Crit. Rev. Oncol. Hematol..

[109] Sharp M., Corp D. KEYTRUDA® (Pembrolizumab) Injection, for Intravenous Use Initial U.S. Approval: 2014. https://www.merck.com/product/usa/pi_circulars/k/keytruda/keytruda_pi.pdf.

[110] Spain L., Diem S., Larkin J. (2016). Management of toxicities of immune checkpoint inhibitors. Cancer Treat. Rev..

[111] Larkin J., Chiarion-Sileni V., Gonzalez R., Grob J.J., Cowey C.L., Lao C.D., Schadendorf D., Dummer R., Smylie M., Rutkowski P. (2015). Combined Nivolumab and Ipilimumab or Monotherapy in Untreated Melanoma. N. Engl. J. Med..

[112] Nishijima T.F., Shachar S.S., Nyrop K.A., Muss H.B. (2017). Safety and tolerability of PD-1/PD-l1 inhibitors compared with chemotherapy in patients with advanced cancer: A meta-analysis. Oncologist.

[113] Man J., Ritchie G., Links M., Lord S., Lee C.K. (2018). Treatment-related toxicities of immune checkpoint inhibitors in advanced cancers: A meta-analysis. Asia Pac. J. Clin. Oncol..

[114] Pitt J.M., Vétizou M., Daillère R., Roberti M.P., Yamazaki T., Routy B., Lepage P., Boneca I.G., Chamaillard M., Kroemer G. (2016). Resistance mechanisms to Immune-Checkpoint blockade in cancer: Tumor-Intrinsic and -Extrinsic factors. Immunity.

[115] Adhikaree J., Moreno-Vicente J., Kaur A.P., Jackson A.M., Patel P.M. (2020). Resistance mechanisms and barriers to successful immunotherapy for treating glioblastoma. Cells.

[116] Kvistborg P., Philips D., Kelderman S., Hageman L., Ottensmeier C., Joseph-Pietras D., Welters M.J.P., van der Burg S., Kapiteijn E., Michielin O. (2014). Anti-CTLA-4 therapy broadens the melanoma-reactive CD8+ T cell response. Sci. Transl. Med..

[117] Chongsathidkiet P., Jackson C., Koyama S., Loebel F., Cui X., Farber S.H., Woroniecka K., Elsamadicy A.A., Dechant C.A., Kemeny H.R. (2018). Sequestration of T cells in bone marrow in the setting of glioblastoma and other intracranial tumors. Nat. Med..

[118] Woroniecka K., Chongsathidkiet P., Rhodin K., Kemeny H., Dechant C., Farber S.H., Elsamadicy A.A., Cui X., Koyama S., Jackson C. (2018). T-Cell exhaustion signatures vary with tumor type and are severe in glioblastoma. Clin. Cancer Res..

[119] Brooks W.H., Caldwell H.D., Mortara R.H. (1974). Immune responses in patients with gliomas. Surg. Neurol..

[120] Brooks W.H., Roszman T.L., Rogers A.S. (1976). Impairment of rosette-forming T lymphocytes in patients with primary intracranial tumors. Cancer.

[121] Fecci P.E., Mitchell D.A., Whitesides J.F., Xie W.H., Friedman A.H., Archer G.E., Herndon N.J.E., Bigner D.D., Dranoff G., Sampson J.H. (2006). Increased regulatory T-cell fraction amidst a diminished CD4 compartment explains cellular immune defects in patients with malignant glioma. Cancer Res..

[122] Dunn G.P., Fecci P.E., Curry W.T. (2012). Cancer immunoediting in malignant glioma. Neurosurgery.

[123] Woroniecka K.I., Rhodin K.E., Chongsathidkiet P., Keith K.A., Fecci P.E. (2018). T-cell dysfunction in glioblastoma: Applying a new framework. Clin. Cancer Res..

[124] Preusser M., Lim M., Hafler D.A., Reardon D.A., Sampson J.H. (2015). Prospects of immune checkpoint modulators in the treatment of glioblastoma. Nat. Rev. Neurol..

